# Doppler Ultrasound Findings in Filler-Related Facial Vascular Adverse Events: An International Multicenter Study

**DOI:** 10.3390/diagnostics16111587

**Published:** 2026-05-22

**Authors:** Rosa M. S. Sigrist, Claudia Gonzalez, Leonie Schelke, Ximena Wortsman, Stella Desyatnikova, Fernanda A. Cavallieri, Maria Cristina Chammas

**Affiliations:** 1 Department of Radiology, Hospital das Clínicas, School of Medicine, University of São Paulo, São Paulo 05403-010, Brazil; cristina.chammas@hc.fm.usp.br; 2 Ultrassonando Clinic, São Paulo 04530-001, Brazil; 3 Highly Specialized Ultrasound Center, Bogota 111121, Colombia; claud_gonzalezdiaz@yahoo.com; 4 Department of Dermatology, Erasmus MC University Medical Center, P.O. Box 2040, 3000 CA Rotterdam, The Netherlands; lschelke@outlook.com; 5 Department of Dermatology, Faculty of Medicine, Universidad de Chile, Santiago 8380453, Chile; 6 Department of Dermatology, School of Medicine, Pontificia Universidad Catolica de Chile, Santiago 8331150, Chile; 7 Department of Dermatology and Cutaneous Surgery, Miller School of Medicine, University of Miami, Miami, FL 33136, USA; 8 Institute for Diagnostic Imaging and Research of the Skin and Soft Tissues, Santiago 7591018, Chile; 9 The Stella Center for Facial Plastic Surgery, Seattle, WA 98101, USA; stella@doctorstella.com; 10 Cavallieri Clinic, Rio de Janeiro 22440-040, Brazil; nandacavallieri@gmail.com

**Keywords:** facial fillers, vascular occlusion, Doppler ultrasound, hyaluronidase, vascular complications, ultrasound guidance

## Abstract

**Background**: Vascular adverse events (VAEs) related to facial filler injections are rare but potentially severe complications. Doppler ultrasound has emerged as an adjunct imaging tool for evaluating vascular compromise; however, Doppler findings in facial VAEs remain insufficiently characterized. **Objectives**: To characterize Doppler ultrasound findings associated with filler-related facial VAEs and to assess whether Doppler patterns differ according to prior hyaluronidase administration. **Methods**: This international multicenter retrospective observational study included 100 patients with clinically diagnosed facial VAEs following filler injections between May 2022 and April 2025. Doppler ultrasound findings were analyzed, including absent flow in perforators and major arteries, compensatory flow, abnormal waveforms, increased peak systolic velocity (PSV), and absence of Doppler abnormalities. Patients were categorized according to hyaluronidase administration prior to ultrasound evaluation. Descriptive statistics and comparative analyses were performed. **Results**: One hundred patients (median age, 38 years; IQR: 30–50; 88 women) were evaluated. The most frequent Doppler ultrasound findings were absent flow in perforators (42%) and major arteries (35%), followed by compensatory flow (26%), string sign (18%), flow diversion (16%), and increased peak systolic velocity (16%). No Doppler abnormalities were observed in 12% of cases, while tardus–parvus (9%) and staccato waveform (8%) were less frequent. Doppler ultrasound findings did not differ significantly between patients who received hyaluronidase before imaging and those who did not (all *p* > 0.05). The dose of hyaluronidase varied substantially. Livedo reticularis, blanching, and pain were the most common clinical findings. Central facial arterial territories, particularly the perioral, nasolabial fold, nasal, and glabellar regions, were most commonly involved. **Conclusions**: Filler-related facial VAEs show recognizable Doppler ultrasound patterns, and the identification of these patterns may improve localization of vascular occlusion and support ultrasound-guided hyaluronidase administration, potentially enabling more targeted delivery with lower doses.

## 1. Introduction

The use of injectable fillers has increased substantially worldwide over the past decade. In 2023, more than 19.1 million nonsurgical aesthetic procedures were performed globally, with hyaluronic acid being the most commonly used filler [1]. Although vascular adverse events (VAEs) related to filler injections are relatively uncommon, occurring in approximately 1 in 6600 procedures (0.015%), the high procedural volume makes these complications clinically relevant and likely underreported [2].

Filler-induced VAEs may result in severe and potentially irreversible complications if not promptly identified and managed, including skin necrosis, visual impairment or blindness, and cerebrovascular events [2,3,4,5]. For this reason, they are considered clinical emergencies requiring prompt recognition and immediate management. Early diagnosis and precise localization of vascular compromise are critical to optimize treatment and minimize tissue damage.

While the diagnosis of VAEs remains primarily clinical, Doppler ultrasound has become an increasingly important adjunctive tool [6,7,8]. It enables confirmation of vascular obstruction; identification of the affected vessel; assessment of the extent of ischemia; guidance for targeted hyaluronidase administration, an enzyme that degrades hyaluronic acid and is used in the management of these complications; and monitoring of therapeutic response.

Unlike the carotid or peripheral arteries, where Doppler ultrasound criteria for stenosis and occlusion are well established [9,10], the facial vasculature is highly complex and extensively anastomosed [11,12,13]. As a result, standardized Doppler ultrasound findings associated with facial VAEs remain insufficiently characterized in the literature.

In this context, the present study aimed to characterize the most frequent Doppler ultrasound findings in filler-related facial vascular adverse events. We also assessed whether Doppler findings differed between patients who had received hyaluronidase prior to ultrasound evaluation and those who had not and described the main clinical findings and arterial territories involved.

## 2. Materials and Methods

### 2.1. Study Design

The study was conducted using retrospective, de-identified data shared by participating centers under the coordination of the lead institution. Due to the retrospective nature of the study, the requirement for written informed consent was waived. Written informed consent was obtained for the publication of clinical images when applicable. All procedures were conducted in accordance with the Declaration of Helsinki. The study was approved by the Ethics Committee of the Hospital das Clínicas, Faculty of Medicine, University of São Paulo (CAAE 94746725.7.0000.0068, approved date: 4 February 2026).

### 2.2. Participants

Potentially eligible patients were identified retrospectively at six collaborating institutions, including four radiology centers, one dermatology center, and one plastic surgery center, located in Brazil, Chile, Colombia, the Netherlands, and the United States. Patients were identified between May 2022 and April 2025.

Inclusion criteria were clinically diagnosed facial VAEs following aesthetic filler injections in patients who underwent Doppler ultrasound evaluation. Clinical diagnosis was based on characteristic signs of vascular compromise occurring during or shortly after filler injection, consistent with the typical temporal progression of cutaneous ischemia. Findings included pain disproportionate to the procedure, delayed capillary refill, blanching, livedo reticularis, pustules, ecchymosis, coagulative necrosis, or eschar [13,14], distributed in patterns compatible with known facial arterial territories. No exclusion criteria were applied. All eligible patients referred for Doppler ultrasound during the study period were included, resulting in a consecutive case series. Sample size was determined by the number of eligible patients available during the study period. No overlap with previously published cohorts was present.

### 2.3. Ultrasound Protocol

All patients underwent high-frequency ultrasound examinations using linear-array transducers operating at frequencies ranging from 18 to 24 MHz, following published dermatologic ultrasound protocols [15]. Patients were in the supine or slightly reclined position. Color Doppler, power Doppler, and spectral Doppler imaging were performed in all cases, with Doppler parameters optimized for detection of low-velocity flow in superficial facial vessels. The velocity scale was set at approximately 3 cm/s and adjusted as needed to optimize visualization of slow flow in small-caliber arteries and perforators, with corresponding adjustment of pulse repetition frequency and wall filter settings. Abundant coupling gel was used, and minimal transducer pressure was applied to avoid compression of superficial vessels and soft tissues. Microvascular imaging techniques and/or echoangio software was additionally used in selected cases, particularly to improve detection of low-velocity flow in small-caliber arteries and superficial perforators. These techniques were not available in all centers and were used as complementary tools without altering the classification of Doppler patterns. Ultrasound systems included Logiq E10 and Venue (GE Healthcare), Affiniti 70 and EPIQ (Philips Healthcare), and Aplio 200 (Canon Medical Systems).

Given the emergency nature of filler-related vascular adverse events, hyaluronidase is typically administered promptly after recognition of clinical signs, without delaying treatment for imaging confirmation. Across participating centers, Doppler ultrasound examinations were most commonly performed within 1–15 days after the vascular event, frequently within the first few days and often on the same day as treatment, reflecting real-world clinical practice.

### 2.4. Image Acquisition and Interpretation

Ultrasound examinations were performed by experienced operators, including four radiologists, one plastic surgeon, and one aesthetic physician, all with at least 10 years of experience in aesthetic and vascular ultrasound. Examinations were performed as part of routine clinical care, and operators were not blinded to clinical information.

Doppler ultrasound evaluation was directed to the anatomical region corresponding to the clinical signs of vascular compromise identified at presentation, focusing on arteries that supplied the affected territory, with comparison to adjacent or contralateral segments when relevant. Blood flow was compared with the contralateral and/or peripheral healthy vascular region.

Image interpretation was based on perforators and major arteries. Perforators were identified as small arterial branches directed toward the skin surface. In this study, the term “perforators” was used descriptively to denote these superficially oriented arterial branches visualized on Doppler imaging, recognizing that ultrasound alone does not always allow definitive classification according to surgical anatomy. Major arteries were defined as named facial arteries consistently described in anatomical literature and identifiable on Doppler ultrasound, corresponding to the main axial arterial pathways supplying facial tissues (e.g., facial, angular, infraorbital, supratrochlear, supraorbital, dorsal nasal, and superficial temporal arteries).

Doppler findings were adapted from hemodynamic patterns commonly used in the evaluation of arterial occlusion and stenosis in carotid, cerebral, and peripheral arteries [9,10,16,17,18,19,20,21,22,23] and were assessed based on qualitative and comparative analysis of color Doppler signal and spectral waveform within the evaluated arterial territories. The main patterns evaluated included absent flow in perforators, absent flow in major arteries, compensatory flow, flow diversion to branching vessels, string sign, staccato waveform, tardus–parvus waveform, increased peak systolic velocity, and absence of Doppler abnormalities.

Absent flow in major arteries or in perforators was defined as the complete absence of a color Doppler signal within the arteries despite optimization of Doppler parameters.

Compensatory flow was defined as relatively increased color Doppler signal surrounding an area of absent or markedly reduced flow, typically characterized by a central region with minimal or absent vascular signal (“silent area”) and increased signal in adjacent arterial pathways, suggesting collateral redistribution of blood flow.

Flow diversion to branching vessels was defined as the deviation of blood flow toward alternative arterial branches, bypassing an area of absent flow.

The string sign was defined as markedly reduced vessel caliber associated with a thin linear color Doppler signal, indicating reduced luminal diameter and flow.

The staccato waveform was defined as a high-resistance spectral Doppler pattern with a short systolic spike followed by minimal diastolic flow, reflecting reduced antegrade flow distal to the measurement site.

The tardus–parvus waveform was defined as a damped monophasic waveform with a delayed systolic upstroke and reduced peak velocity, associated with a resistive index (RI) < 0.4, suggesting proximal flow limitation.

Increased peak systolic velocity (PSV) was defined as a PSV higher than the contralateral artery or adjacent arterial segments within the same vascular territory or an increase greater than 50% relative to the proximal segment.

Absence of Doppler abnormalities was defined as preserved color Doppler signal and spectral waveform without evidence of abnormal flow patterns in the evaluated arterial territory.

For data collection, participating centers completed a standardized spreadsheet developed by the coordinating institution, including predefined Doppler variables, clinical features, anatomical territories involved, and information regarding hyaluronidase administration prior to imaging. Each investigator recorded findings based on the original ultrasound examination and clinical documentation. The completed datasets were compiled and organized by the coordinating center for analysis.

### 2.5. Subgroup Analysis

Patients were categorized according to whether hyaluronidase was administered prior to Doppler ultrasound evaluation (yes vs. no). Comparisons of Doppler ultrasound findings between groups were performed as an exploratory analysis in this retrospective cohort using chi-square or Fisher’s exact tests, as appropriate.

### 2.6. Statistical Analysis

Data were summarized using descriptive statistics, with categorical variables expressed as frequencies and percentages and continuous variables reported as medians and interquartile ranges. Associations between categorical variables were assessed using the chi-square test or Fisher’s exact test, as appropriate. The strength of association between binary variables was quantified using the phi (φ) correlation coefficient. A two-sided *p*-value < 0.05 was considered statistically significant. Confidence intervals (95% CIs) were calculated for key proportions and measures of association to provide estimates of precision.

To account for multiple testing in exploratory analyses of associations between Doppler ultrasound findings, *p*-values were adjusted using the Benjamini–Hochberg procedure to control the false discovery rate (FDR). The correction was applied to the family of pairwise associations between Doppler variables evaluated using phi correlation coefficients. Adjusted *p*-values < 0.05 were considered statistically significant.

Statistical analyses were performed using R version 4.3.2 (R Foundation for Statistical Computing, Vienna, Austria) with RStudio version 2023.09.1 (RStudio PBC, Boston, MA, USA). No formal sample size or power calculation was performed due to the retrospective observational design of the study.

## 3. Results

### 3.1. Patient Characteristics

A total of 100 patients with clinically diagnosed filler-related facial vascular adverse events were included. No patients were excluded, as all referred cases met the inclusion criteria. Baseline demographic and procedural characteristics are summarized in Table 1.

### 3.2. Doppler Ultrasound Findings

**Figure 1:**
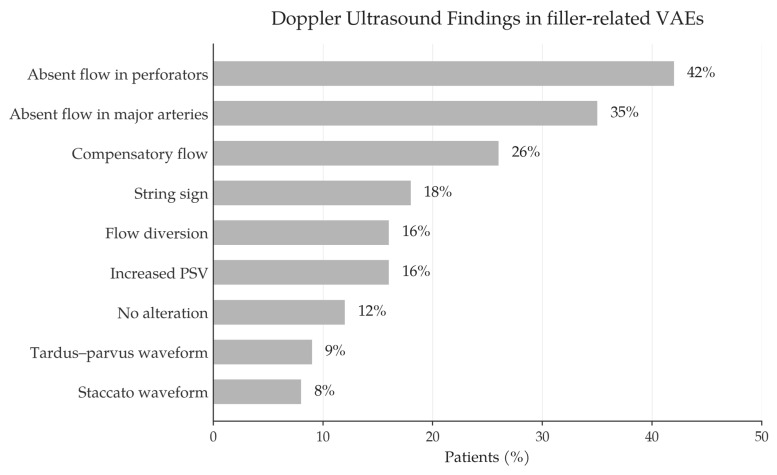
Doppler ultrasound findings in filler-related vascular adverse events (VAEs). Doppler findings were not mutually exclusive, and more than one finding could be present in the same patient.

**Figure 2:**
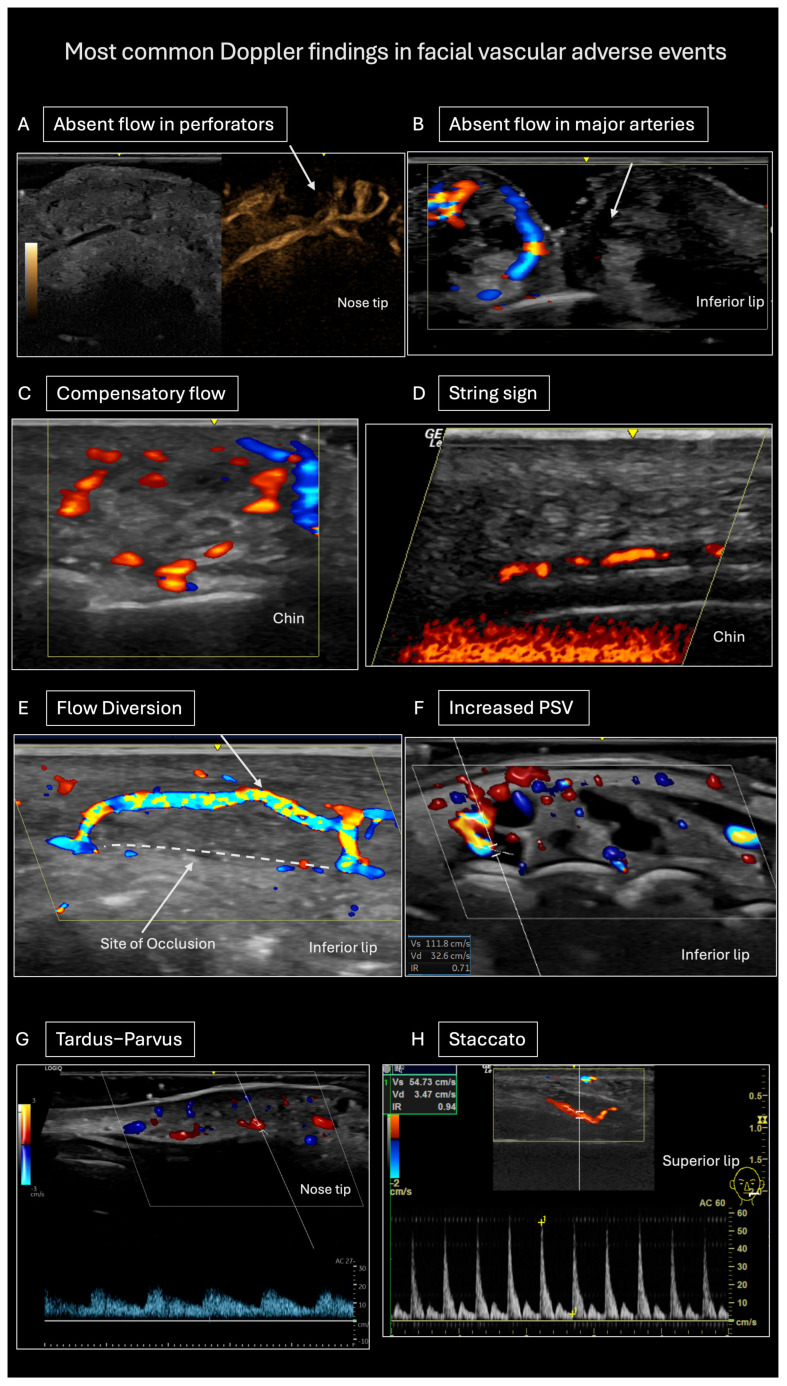
Images of Doppler ultrasound findings in filler-related VAEs. (**A**) B-Flow US image of a 45-year-old female participant 7 days after hyaluronic acid in the nose showing absent flow of perforators on the tip of the nose. (**B**) Color Doppler US images showing absent flow in the inferior labial artery in a 25-year-old female participant one hour after the injection of hyaluronic acid in the lower lip. (**C**) Color Doppler ultrasound images in a 30-year-old male participant, who received a hyaluronic acid injection in the chin 17 h prior to the exam, demonstrate compensatory flow surrounding a region without detectable Doppler signal (silent area). (**D**) Power Doppler ultrasound of a 55-year-old female participant who received a hyaluronic acid injection in the lower lip 48 h prior to the ultrasound examination shows a string sign (filiform flow) in the right labiomental artery. (**E**) Color Doppler US in a 22-year-old female injected with hyaluronic acid in the lips demonstrates occlusion of the inferior labial artery, with flow diversion to a branching vessel bypassing the site of occlusion. (**F**) PW Doppler of the inferior labial artery in a 66-year-old female participant demonstrates increased PSV in the inferior labial artery, which is surrounded by a hyaluronic acid deposit. (**G**) PW Doppler of a 33-year-old male injected with hyaluronic acid in the nose demonstrating a damped flow pattern (tardus–parvus waveform) in the nasal artery. (**H**) PW Doppler of a 42-year-old female injected with hyaluronic acid demonstrates a high resistive index in the superior labial artery, corresponding to a staccato waveform.

On Doppler ultrasound evaluation, the most frequent findings were absent flow in perforators (42%, 95% CI: 33–52%) and absent flow in major arteries (35%, 95% CI: 26–45%). Additional Doppler patterns included compensatory flow (26%, 95% CI: 18–35%), string sign (18%, 95% CI: 12–27%), flow diversion to branching vessels (16%, 95% CI: 10–24%), and increased peak systolic velocity (16%, 95% CI: 10–24%). In a subset of cases, no Doppler abnormalities were detected (12%, 95% CI: 7–20%). Less frequent waveform abnormalities included tardus–parvus waveform (9%, 95% CI: 5–16%) and staccato waveform (8%, 95% CI: 4–15%). Because Doppler patterns may coexist within the same vascular territory, more than one finding could be observed in a single patient. The distribution of Doppler ultrasound findings is summarized in Figure 1, with representative imaging examples shown in Figure 2.

A moderate association was observed between absent flow in perforator vessels and compensatory flow (Φ = 0.37, *p* < 0.001), and this association remained statistically significant after false discovery rate correction for multiple comparisons (adjusted *p* = 0.017). No other clinically meaningful associations remained significant after FDR adjustment.

### 3.3. Doppler Findings According to Prior Hyaluronidase Use

Comparisons of Doppler ultrasound findings between patients who received hyaluronidase prior to imaging and those who did not are summarized in Table 2. Among patients who received hyaluronidase, administered doses varied widely.

In this exploratory analysis, no statistically significant differences in the distribution of Doppler findings were detected between patients who had received hyaluronidase before ultrasound evaluation and those who had not (all *p* > 0.05). Comparisons between groups are presented in Table 3.

### 3.4. Clinical Signs and Associations

**Figure 3:**
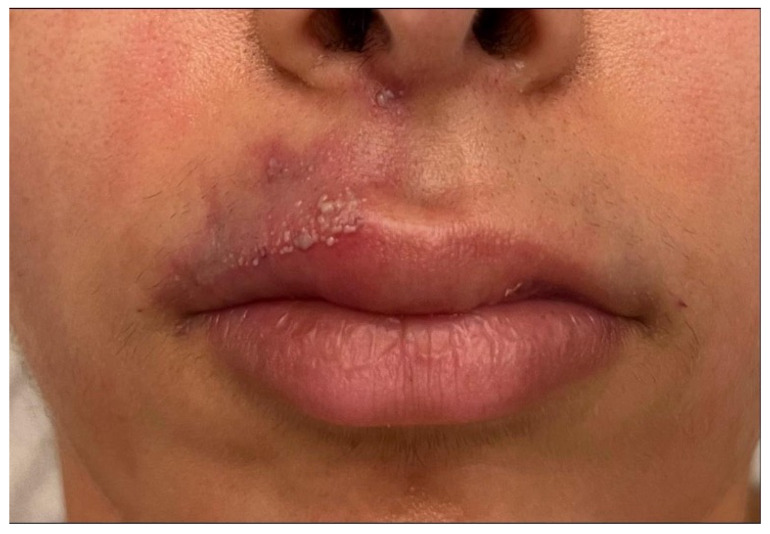
Clinical photograph in a 22-year-old woman with a filler-related facial vascular adverse event following hyaluronic acid injection for lip augmentation. The image, obtained 4 days after injection, shows livedo reticularis and pustules involving the perioral region.

**Figure 4:**
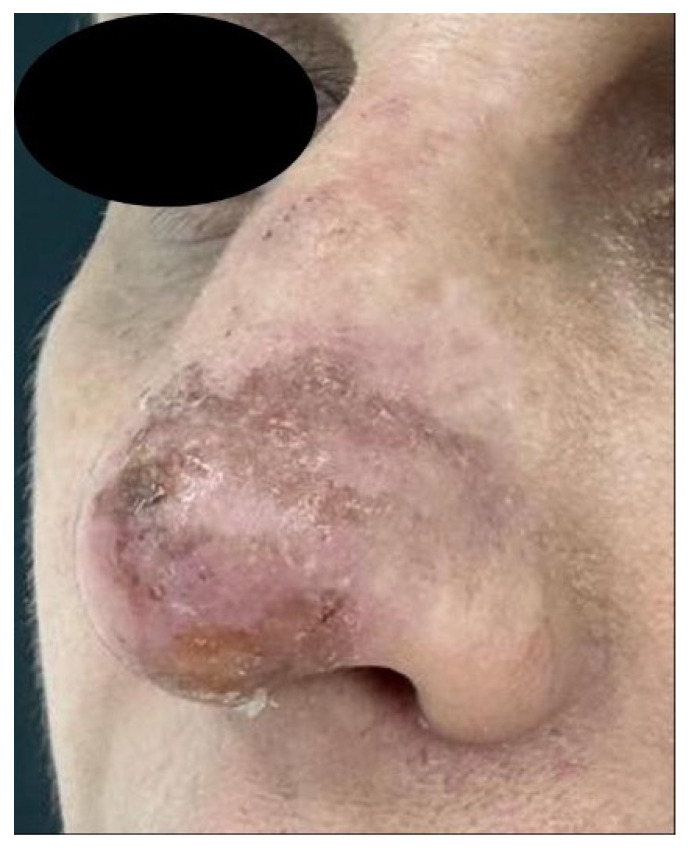
Clinical photograph in a 45-year-old woman with a filler-related facial vascular adverse event following hyaluronic acid injection in the nose. The image, obtained 7 days after injection, shows nasal eschar.

All patients included in the study had a clinical diagnosis of VAEs following filler injection, established based on characteristic clinical signs. Among these, livedo reticularis (Figure 3) was the most frequent finding, observed in 46 of 100 patients (46%; 95% CI: 37–56%), followed by blanching in 38 of 100 (38%; 95% CI: 29–48%) and pain in 25 of 100 (25%; 95% CI: 18–34%). Pustules were observed in 20 of 100 patients (20%; 95% CI: 13–29%) (Figure 4) and ecchymosis in 16 of 100 (16%; 95% CI: 10–24%), whereas coagulative necrosis was present in 3 of 100 (3%; 95% CI: 1–8%) and eschar in 1 of 100 (1%; 95% CI: 0.2–5%) (Figure 4). These clinically established cases subsequently underwent Doppler ultrasound to characterize vascular involvement. A statistically significant association was observed between livedo reticularis and reported pain (Φ = 0.25, 95% CI: 0.06–0.43; *p* = 0.019), indicating that these clinical features tend to co-occur in patients with VAE.

### 3.5. Arterial Territories Involved

**Figure 5:**
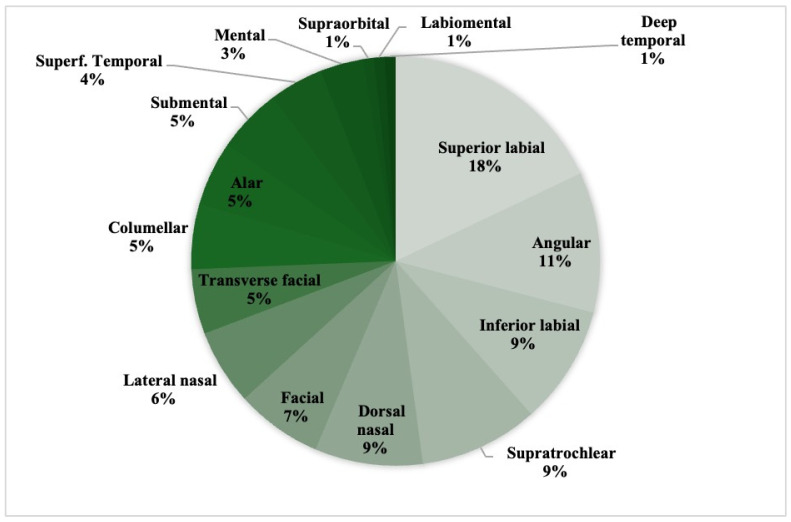
Distribution of arterial involvement in filler-related vascular adverse events (VAEs). Percentages represent the relative frequency of each affected artery based on the total number of arterial involvements identified (n = 117) across the 100 patients. More than one artery could be involved in a single patient. Percentages are rounded to the nearest whole number and may not sum to exactly 100% due to rounding.

Arterial involvement was most frequently observed in central facial territories, including the perioral, nasolabial fold, nasal, and glabellar regions. The perioral arteries (superior and inferior labial) were the most commonly affected, followed by the angular, dorsal nasal, and supratrochlear arteries. In several cases, more than one artery was involved within the same patient, reflecting the anatomical continuity and extensive anastomotic network of the facial vasculature. Figure 5 shows the distribution of arterial involvement in VAEs based on the total number of arterial involvements identified across the study sample, allowing characterization of the relative frequency of each affected arterial territory.

## 4. Discussion

This international multicenter retrospective study demonstrates that filler-related vascular occlusions present consistent and recognizable Doppler ultrasound patterns that reflect the complex organization of the facial arterial network. The predominant finding was absent flow in perforator vessels, frequently associated with compensatory flow, followed by absent flow in major arteries. It should be kept in mind that most patients had received prior hyaluronidase injections before the ultrasound examination; therefore, the original blood flow characteristics of the vascular occlusion may have been modified, such as the opening of the main vessels. However, the perforators or microvascular bed can still be occluded. Nevertheless, this is the first attempt to characterize the Doppler findings in these cases. By characterizing these Doppler alterations, our results provide a clinically applicable framework for interpreting ultrasound findings in cases of filler-related vascular compromise.

Our findings are supported by previous reports describing Doppler ultrasound alterations in filler-related vascular occlusions, although usually reported as individual observations rather than as a structured range of findings. Prior studies have described absence of blood flow [24,25,26], waveform abnormalities [24,25,26], no alterations [25], and more recently, absence of flow in perforators [6]. Taken together, both our results and prior literature suggest that vascular occlusions may present with multiple Doppler abnormalities, often existing in the same patient, likely reflecting the complex hemodynamic behavior of the facial arterial network.

The arteries of the face are tortuous and extensively anastomosed, with multiple angiosomes, three-dimensional vascular territories comprising skin and underlying tissues supplied by a source artery and its branches, which facilitate collateral recruitment and redistribution of blood flow [27,28]. This anatomical organization helps explain the wide range of Doppler ultrasound findings, including patients with clear clinical signs of VAEs but no detectable Doppler abnormalities. In these cases, vascular compromise may involve small arterial branches, arterioles, or capillaries [3], which may not be readily detected with conventional Doppler techniques. In addition, collateral perfusion through direct and choke vessels, which are small-caliber anastomotic connections between adjacent angiosomes that can dilate under ischemic conditions, may partially preserve detectable flow despite underlying ischemia [29,30]. Interestingly, to date, the usual detection threshold of velocity in the devices is 2 cm/s; therefore, minor arteries may not show flow. Furthermore, most patients without Doppler abnormalities had received hyaluronidase before ultrasound, which may reflect restoration of blood flow prior to imaging. Nevertheless, the perforators and microvasculature occlusion seem difficult to treat solely clinically, and the patients benefit from the better localization of abnormal flow via the color Doppler ultrasound examination.

From a physiological perspective, absence of detectable flow in perforators may reflect distal compromise of angiosome perfusion, potentially caused by distal embolization of filler material after indirect extraluminal inoculation [3] or by severe vasospasm leading to critical caliber reduction of small vessels [6,7]. In contrast, absence of flow in a major artery is more suggestive of complete arterial occlusion, most plausibly related to direct intraluminal filler injection. The association between absent flow in perforators and compensatory flow supports the concept that localized perfusion impairment frequently triggers collateral recruitment through adjacent vascular territories, analogous to mechanisms described in cerebral circulation [16]. Increased PSV, a well-established marker of hemodynamically significant stenosis in carotid [9,21] and peripheral artery disease [10,31], was less frequently observed in facial arteries, likely reflecting technical limitations inherent to facial Doppler imaging, including small vessel caliber, tortuosity, suboptimal insonation angles, and Doppler settings optimized for low-flow and microcirculatory assessment.

Doppler findings did not differ significantly between patients who had received hyaluronidase prior to ultrasound evaluation and those who had not. Given the small size of the untreated group (n = 21), the non-randomized administration of hyaluronidase, and the limited statistical power, the absence of detected differences should be interpreted with caution. Among possible contributors to this lack of detectable difference, most treatments were performed without imaging guidance before referral for ultrasound, meaning that hyaluronidase may not have been delivered precisely to the affected vascular areas. Previous studies have shown that ultrasound-guided hyaluronidase allows more precise targeting and is associated with improved clinical outcomes [6,32,33]. In addition, the dose of hyaluronidase administered prior to ultrasound evaluation varied substantially, likely reflecting uncertainty about the exact location of vascular compromise. Taken together, these findings suggest that precise localization of the affected vascular territory may be more relevant than dose alone.

The clinical findings observed in this cohort are consistent with the natural history of filler-related vascular occlusion, which typically begins with pain or blanching and may progress to livedo reticularis and, in more advanced stages, tissue necrosis if untreated [13,34]. The predominance of early manifestations suggests that most patients were evaluated during an initial phase of vascular compromise. The frequent association between livedo reticularis and pain supports this interpretation and indicates that Doppler ultrasound was often performed within a window in which hemodynamic alterations can still be detected, allowing identification of the affected vascular territory and supporting timely management.

Vascular involvement most often affected central facial regions, including the perioral, nasolabial fold, nasal areas, and the glabella, a distribution that is consistent with previous studies describing a higher frequency of vascular complications in these anatomically complex territories [30,34,35,36,37]. These territories are anatomically important because of their extensive vascular connections between the external and internal carotid systems [38]. As a result, vascular compromise in these areas carries a higher risk of serious complications, such as visual loss and cerebrovascular events. Recognition of these patterns may assist clinicians in prioritizing prompt vascular assessment when symptoms involve central facial regions.

This study has limitations inherent to its retrospective observational design. Ultrasound examinations were performed as part of routine clinical care without blinding to clinical findings, which may have introduced observer bias, and the absence of a control group limits comparison with other post-procedural conditions. The multicenter design involved different ultrasound systems and acquisition settings; although operational definitions of Doppler findings were predefined by the coordinating center and all examinations were performed by experienced clinicians, including four radiologists, with established expertise in Doppler ultrasound (Section 2.4), formal interobserver agreement was not assessed, and future prospective studies with centralized image review may further support reproducibility. This descriptive study was not designed to analyze the temporal evolution of Doppler findings; quantitative timing intervals (filler injection to symptom onset, symptom onset to hyaluronidase administration, hyaluronidase to ultrasound, and vascular event to ultrasound) were not systematically recorded and could not be analyzed in a standardized fashion. The sample size and exploratory statistical analysis did not allow adjustment for potential confounding factors, and the small size of the untreated subgroup (n = 21) further limits the ability to detect differences between groups. Clinical outcome data (resolution, progression to necrosis, additional interventions) were not uniformly captured, as long-term follow-up was performed by the referring injector rather than by the participating ultrasound centers; a correlation between Doppler findings and clinical outcomes should be addressed in future prospective studies.

## 5. Conclusions

In conclusion, filler-related facial vascular adverse events demonstrate consistent Doppler ultrasound patterns that reflect the complex collateral organization of the facial arterial network. Absent flow, particularly in perforator vessels, was the predominant finding and was moderately associated with compensatory flow, supporting the presence of adaptive hemodynamic responses within compromised vascular territories. Central facial arteries were most frequently involved, and prior hyaluronidase administration was not associated with differences in Doppler findings. These findings highlight the clinical relevance of Doppler ultrasound for identifying hemodynamic alterations and localizing the most probable site of vascular occlusion, supporting ultrasound-guided hyaluronidase administration and potentially enabling more targeted delivery with lower doses.

## Figures and Tables

**Table 1:** Baseline demographic and procedural characteristics of patients with filler-related vascular adverse events.

Variable	N = 100
**Age, years (n = 99) ^1^**	38 (30, 50) **^2^**
**Sex**	
Female	88 (88%)
Male	12 (12%)
**Filler type**	
Hyaluronic acid	98 (98%)
Calcium hydroxyapatite	2 (2%)
**Hyaluronidase before ultrasound**	
Yes	79 (79%)
No	21 (21%)

^1^ Age was missing for one patient. ^2^ Median (1st quartile, 3rd quartile).

**Table 2:** Hyaluronidase use and administered doses prior to Doppler ultrasound evaluation.

	Category	n	%
Hyaluronidase dose	No hyaluronidase	21	21%
	1–1500 IU	26	26%
	1501–3000 IU	11	11%
	3001–4500 IU	9	9%
	>4500 IU	24	24%
	Dose not recorded	9	9%

IU: international units.

**Table 3:** Doppler ultrasound findings according to prior hyaluronidase use. Comparisons between groups are presented. Doppler findings were not mutually exclusive, and more than one finding could be present in the same patient.

	Overall	No	Yes	
Doppler Finding	N = 100 ^1^	N = 21	N = 79	*p*-Value ^2^
Absent flow in perforator	42 (42%)	10 (48%)	32 (41%)	0.6
Absent flow in major artery	35 (35%)	5 (24%)	30 (38%)	0.3
Compensatory flow	26 (26%)	9 (43%)	17 (22%)	0.056
String sign	18 (18%)	4 (19%)	14 (18%)	>0.9
Flow diversion	16 (16%)	6 (29%)	10 (13%)	0.1
Increased PSV	16 (16%)	2 (9.5%)	14 (18%)	0.5
No alteration	12 (12%)	2 (9.5%)	10 (13%)	>0.9
Tardus–parvus waveform	9 (9.0%)	3 (14%)	6 (7.6%)	0.4
Staccato waveform	8 (8.0%)	1 (4.8%)	7 (8.9%)	>0.9

^1^ n (%), ^2^ Fisher’s exact test.

## Data Availability

Dataset available on request from the authors.

## Abbreviations

The following abbreviations are used in this manuscript:

VAEs	Vascular adverse events
PSV	Peak systolic velocity
